# Organic acid blend boosts butyrate and limits pathogens in an exploratory *ex vivo* chicken caecal model

**DOI:** 10.1016/j.psj.2026.107317

**Published:** 2026-06-18

**Authors:** Ana-Maria Imbrea, Igori Balta, Lavinia Stef, Todd Callaway, Ioan Pet, Nicolae Corcionivoschi

**Affiliations:** a Doctoral School “Engineering of Vegetable and Animal Resources”, University of Life Sciences “King Mihai I” from Timişoara, Calea Aradului 119, 300645 Timişoara, Romania; b Faculty of Bioengineering of Animal Resources, University of Life Sciences King Mihai I from Timisoara, 300645 Timisoara, Romania; c Department of Animal and Dairy Science, University of Georgia, Athens, GA, USA; d Bacteriology Branch, Veterinary Sciences Division, Agri-Food and Biosciences Institute, Belfast BT4 3SD, Northern Ireland, UK; e Academy of Romanian Scientists, Ilfov Street, No. 3, 050044 Bucharest, Romania

**Keywords:** Organic acid blend, *Faecalibacterium prausnitzii*, *Salmonella* typhimurium, *Campylobacter jejuni*, Chicken caeca

## Abstract

Organic acid blends are being explored as alternatives to antibiotics in poultry production because of their antimicrobial and microbiota-modulating properties. This study examined whether a subinhibitory concentration of AuraShield could stimulate *Faecalibacterium prausnitzii*-associated butyrate production while reducing colonisation by *Salmonella enterica* serovar Typhimurium and *Campylobacter jejuni* in an *ex vivo* chicken caecal model. Minimum inhibitory and bactericidal concentration assays identified 0.125% AuraShield as a subinhibitory dose. In whole caeca incubated for 24 h under microaerophilic conditions, AuraShield promoted *F. prausnitzii* growth and increased butyrate production. Co-incubation with *F. prausnitzii* reduced pathogen loads, and this effect was further enhanced by AuraShield. The treatment also reduced caecal expression of the inflammatory chemokines *CXCLi1* and *CXCLi2*, as well as the oxidative stress marker *ROMO*1. In complementary co-culture experiments, AuraShield increased *butCo*A expression and stimulated butyrate release by *F. prausnitzii* in a dose-dependent manner. Comparative experiments with inulin showed that AuraShield promoted butyrate production and reduced pathogen growth at levels comparable to, or greater than, those of the prebiotic control. Overall, these findings suggest that AuraShield may support beneficial bacterial activity under *ex vivo* conditions. Further *in vivo* studies are needed to confirm its potential under field conditions.

## Introduction

*Campylobacter jejuni*, a major foodborne pathogen, and *Salmonella enterica*, particularly serovar Typhimurium, a major enteric pathogen, have poultry acting as a major reservoir for contamination during processing and along the food chain, thereby increasing the risk of human infection (Sharma et al., 2025). As a result, controlling *Campylobacter* and *Salmonella* colonisation in poultry caeca is a major priority for improving flock health, reducing bacterial transmission, and enhancing food safety (Ghareeb et al., 2013). These pathogens impose a substantial economic burden on the global poultry industry by affecting production efficiency, flock management, food safety, and public health (Korver, 2023). *Faecalibacterium prausnitzii* is a beneficial gut bacterium that supports intestinal health by producing butyrate, a short-chain fatty acid that maintains epithelial integrity, reduces inflammation, and strengthens colonisation resistance (Sabater et al., 2026). In the context of enteric infections, its presence is particularly relevant, as increased growth and metabolic activity of *F. prausnitzii* can help limit the establishment of important poultry pathogens such as *Salmonella enterica* serovar Typhimurium and *Campylobacter jejuni* (Ahmed et al., 2026). By improving the gut environment and promoting protective microbial interactions, *F. prausnitzii* may reduce pathogen colonisation and help modulate host inflammatory and oxidative stress responses, making it a promising target for microbiota-based strategies in poultry health management (Ferrocino et al., 2023).

Organic acids can be effective antimicrobials that reduce the presence of pathogens in broilers (e.g., *Salmonella* and *Campylobacter*) (Yoon et al., 2024), and when used as blends, can effectively ameliorate the impact of necrotic enteritis in broilers and improve intestinal health (Kumar et al., 2022). However, identifying optimal incorporation dosages remains a challenge, and using *ex vivo* models can help overcome these impediments to provide efficient solutions (Patil et al., 2025). Organic acids appear to play a prebiotic role in farmed animals, providing health benefits to the host gut microbiome (Wei et al., 2021)(Waghmare et al., 2025). There is extensive evidence to state that organic acids improve gut development, nutrient absorption, reduce oxidative stress and improve the immunity in poultry (Ebeid and Al-Homidan, 2022). This effect expands cross species as blends of organic acids (e.g. citric acid and malic acid) have significantly enhanced F. prausnitzii growth and butyrate secretion in a shrimp infection model of Vibrio parahaemolyticus (Butucel et al., 2022). AuraShield, a blend of organic acids, demonstrated significant antimicrobial and anti-virulence effects against *S*. Typhimurium in a chicken caeca model, reducing bacterial adhesion, invasion, and growth, while also protecting host cells from cytotoxicity and inflammation at a subinhibitory concentration of 0.25% (Marcu et al., 2026a; Marcu et al., 2026b). Similar blends of organic acids were previously shown to have antimicrobial and antipathogenic effects against *C. jejuni* RC039 at subinhibitory concentrations above 0.1% (Sima et al., 2018).

There is now clear evidence that blends of organic acids yield better results than using a single organic acid alone (Polycarpo et al., 2017). Hence, studying and classifying novel blends of natural antimicrobials (e.g., organic acids) is challenging because on-field applications are sometimes inefficient without appropriate *in vitro* or *in vivo* models. Using *ex vivo* models, with tissue obtained from live animals and maintained under optimal conditions that mimic natural conditions, will significantly shorten the route to practical experimentation and reduce the need for live-animal work (Kapila et al., 2017). Moreover, the *ex vivo* models will provide an opportunity to study the efficacy of dietary supplements, such as blends of organic acids, and their role during bacterial infection and colonisation in poultry (Imbrea et al., 2025). They have been described as improving poultry performance by altering the microbiome, an effect that depends on the product composition and the poultry's health status (Dittoe et al., 2018). Additionally, they can reduce the presence of enteric pathogens and support beneficial bacteria, thereby improving gut health and colonisation resistance (Adil et al., 2010).

Based on this previous evidence, we hypothesised that blends of organic acids can enhance the activity of host-resident beneficial bacteria, such as *Faecalibacterium prausnitzii*, and, in combination with these probiotic-like bacteria, exert an enhanced anti-pathogenic effect. To test this hypothesis, we used an *ex vivo* poultry caecal infection model, supported by a conventional bacterial co-culture model. Specifically, we have sought to gather data on whether a subinhibitory concentration of the organic acid blend AuraShield can promote the growth and butyrate-producing activity of *Faecalibacterium prausnitzii*, while reducing colonisation by *Salmonella* Typhimurium and *Campylobacter jejuni* and modulating host inflammatory responses. This hypothesis was intended as a preliminary mechanistic question under controlled *ex vivo* conditions, rather than a direct prediction of field performance in live birds. Accordingly, the study was designed to test whether AuraShield could influence pathogen recovery and host-associated markers under standardised conditions that minimise confounding effects from the resident microbiota.

## Materials and methods

### Bacterial strains, growth conditions and organic acid blend

*C. jejuni* RC039 (laboratory collection) was grown on Mueller-Hinton Agar Base (Oxoid Ltd., United Kingdom) supplemented with 5% (vol/vol) defibrinated horse blood (Aquilant Scientific N.I.) under microaerophilic conditions (41.5 °C in 85% N_2_, 5% O_2_, and 10% CO_2_) in a Don Whitley MACS-VA500 microaerophilic workstation (Davidson & Hardy Ltd., United Kingdom) for 48 h. *Salmonella enterica* serovar Typhimurium SE10/72 (laboratory stock) was resuscitated by adding one bead to tryptone soy broth (Oxoid, Basingstoke, UK) plus 0·6% (w/v) yeast extract (Oxoid, Basingstoke, UK) and incubating at 37 °C for 24h. Working cultures were maintained on TSAYE slants at 4 °C. Working cultures were prepared by inoculating Brucella Broth (BB) with a loopful of culture from a slope and incubating at 37 °C for an additional 24h. The antimicrobial blend, AuraShield (As) (5% maltodextrin, 1% sodium chloride, 42% citric acid, 18% sodium citrate, 10% silica, 12% malic acid, 9% citrus extract, and 3% olive extract (w/w) was provided by Environtech Dublin, Ireland.

### Determination of minimum inhibitory concentration (MIC) and minimum bactericidal concentration (MBC)

The two-fold tube dilution method was used to determine the lowest concentration of AuraShield that inhibited bacterial growth (MIC) and the lowest concentration that induced bacterial death (MBC) was evaluated (Sima et al., 2018; Marcu et al., 2026a). AuraShield was diluted (8% to 0.015625% v/v) in TSAYE media. *S*. Typhimurium SE10/72 was harvested by centrifugation, washed twice in PBS, resuspended in TSAYE media, and diluted to 1 × 10^6^ CFU/mL. Non-inoculated bijou (5 mL) tubes containing the same growth medium were used as negative controls, whilst TSAYE tubes without AuraShield were inoculated with individual bacterial cultures as positive controls. *S. Typhimurium* cultures were incubated at 37 °C for 24 h. Tubes that did not show visible growth were considered to be above the MIC. One hundred millilitres were taken from each tube for inoculation, then incubated at 37 °C for 24 h on TSAYE Agar Base (Preston; Oxoid Ltd., United Kingdom). For *C. jejuni*, Aurashield was diluted in Mueller-Hinton broth (MHB), and individual overnight bacterial cultures were harvested, washed with PBS, and diluted to approximately 1 × 10^6^ CFU/mL in MHB. Each tube was inoculated with approximately 5 × 10^5^ CFU/mL of the bacterial culture. The tubes were incubated at 41.5 °C for 48 h. One hundred millilitres were taken from tubes showing no growth and inoculated onto MHA plates; the highest dilution with no microbial growth was considered the MBC. Each assay was repeated three times for each strain. To determine the subinhibitory concentrations, the two pathogens were exposed to different concentrations of the antimicrobial. Following determination of the minimum inhibitory concentration (MIC) of AuraShield under the same experimental conditions, the subinhibitory concentration (0.125%) was selected as a fraction of that MIC to ensure it did not directly inhibit bacterial growth (e.g., 1/2 MIC).

### Ex vivo incubation model

The chicken caeca were prepared as previously described (Marcu et al., 2026a), with additional modifications to enable co-incubation of *Faecalibacterium prausnitzii, Salmonella enterica* serovar Typhimurium SE10/72 and *Campylobacter* jejuni RC039 (all from laboratory stocks). The caeca (n = 3) were obtained from euthanised Ross 308 broilers (ethics committee approval 601 from 14/10/2025 from USV Timișoara), placed in DMEM (Dulbecco Modified Eagle Medium) (Lonza, Analab Ltd., United Kingdom), and transported on ice for immediate preparation (within 60 min) (Fig. 1.1). The caeca were obtained from a flock of broiler chickens that had been tested and declared *Salmonella-* and *Campylobacter*-negative prior to slaughter. In laboratory-sterile settings, the caecal content was removed, and the empty caecal tonsils were gently washed with PBS containing 500 U/mL penicillin and 500 μg/mL streptomycin (3 times for 2 min) to remove resident bacteria. The final wash step (3 times) consisted of PBS only to eliminate any residual antibiotics. As described in Fig. 1.2, the controls included: (2A) DMEM only; (2B) DMEM + 10⁴ *F. prausnitzii*; (2C) DMEM + 10⁴ *S*. Typhimurium; (2D) DMEM + 10⁴ *C. jejuni*; (2E) 10⁴ *F. prausnitzii* + 0.125% As; (2F) 10⁴ *S*. Typhimurium + 0.125% As; and (2G) *C. jejuni* + 0.125% As. Experimental groups (all in DMEM) also included: (2H) 10⁴ *F. prausnitzii* + 10⁴ *S*. Typhimurium; (2I) 10⁴ *F. prausnitzii* + *C. jejuni*; (2J) 10⁴ *F. prausnitzii* + 10⁴ *S*. Typhimurium + 0.125% As; (2L) 10⁴ *F. prausnitzii* + 10⁴ *C. jejuni* + 0.125% As. The caeca were then closed with surgical string, suspended in a flask of DMEM, and incubated at 42 °C under microaerophilic conditions (41.5 °C in 85% N_2_, 5% O_2_, and 10% CO_2_) for 24 h. At the end of the 24 h incubation, the caecal contents were harvested by aspiration using sterile disposable Pasteur pipettes for microbiological analysis.

**Fig. 1:**
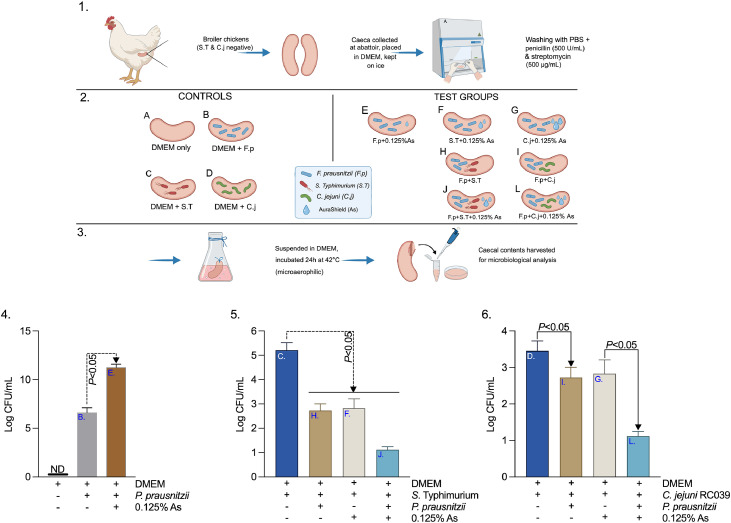
*Ex vivo* chicken caecal co-incubation model to test the impact of 0.125% As on *F. prausnitzii* growth and its exclusion effect against *S*. Typhimurium SE 10/72 and *C. jejuni* RC039 (Figure 1.1). Controls included (Figure 1.2): (2A) DMEM only; (2B) DMEM + 10⁴ *F. prausnitzii*; (2C) DMEM + 10⁴ *S*. Typhimurium; (2D) DMEM + 10⁴ *C. jejuni*; (2E) 10⁴ *F. prausnitzii* + 0.125% As; (2F) 10⁴ *S*. Typhimurium + 0.125% As; and (2G) *C. jejuni* + 0.125% As. Experimental groups also included: (2H) 10⁴ *F. prausnitzii* + 10⁴ *S*. Typhimurium; (2I) 10⁴ *F. prausnitzii* + *C. jejuni*; (2J) 10⁴ *F. prausnitzii* + 10⁴ *S*. Typhimurium + 0.125% As; (2L) 10⁴ *F. prausnitzii* + 10⁴ *C. jejuni* + 0.125% As. All controls and experimental caeca were incubated at 42 °C under microaerophilic conditions for 24 h (Figure 1.3). Figure 1.4 - impact of 0.125% As on *F. prausnitzii* growth; Figure 1.5 - impact of 0.125% As on *S.* Typhimurium during co-incubation with *F. prausnitzii* growth; Figure 1.6 - impact of 0.125% As on *C. jejuni* during co-incubation with *F. prausnitzii*. Data are presented as mean ± SD. Statistical significance was assessed using one‑way ANOVA with Bonferroni’s test for multiple comparisons; *P* < 0.05 was considered statistically significant.

### Quantification of F. prausnitzii, S. Typhimurium SE10/72 and C. jejuni RC039

*F. prausnitzii* quantification was performed as previously described (Butucel et al., 2022). Briefly, caecal contents were used for gDNA extraction using the QIAamp DNA Mini Kit (Qiagen, Manchester, UK) according to the manufacturer’s instructions. The extracted gDNA was stored at −20 °C until required. The 16S rRNA primers used for *F. prausnitzii* quantification were 16SF (ggaggaagaaggtcttcgg) and 16SR (aattccgcctacctctgcact). For the detection of universal bacteria, the primers actcctacgggaggcagcagt (F) and gtattaccgcggctgctggcac (R) were used. The *F. prausnitzii* 16S rRNA gene copies were quantified using the LightCycler 96 (Roche, Burgess Hill, UK) with standard curves generated from 10-fold serial dilutions of amplified *F. prausnitzii* rRNA genes. The relative abundance of *F. prausnitzii* 16S rRNA gene copies was calculated. The relative abundance of target bacterial species with respect to total bacteria was calculated as 2^−ΔCt^ = 2−(Ct of target bacteria − Ct of total bacteria). The limit of detection for qPCR assays was set at 10–100 copies. The experiment was performed in triplicate. *S*. Typhimurium SE10/72 bacteria in the caecal assay were enumerated as previously described (Marcu et al., 2026b). Briefly, 50 μL of content was plated on TSYE agar media containing tergitol (4.6 mL/L), novobiocin (15 mg/L), and cefesulodin (5 mg/L). Plates were incubated at 37 °C for 18 to 22 h, then at room temperature for a further 22 h. *C. jejuni* RC039 were enumerated as previously described (Sima et al., 2018). Briefly, a tenfold dilution (in PBS) of the caecal contents was plated onto CCDA agar (Charcoal Cefoperazone Deoxycholate Agar), and colonies were enumerated after 2 days of incubation at 41.5 °C in microaerophilic conditions. The results were expressed as log CFU/mL.

### Inulin control vs AuraShield

Next, the ex vivo chicken caeca model was used to co-incubate *Faecalibacterium prausnitzii, Salmonella enterica* serovar Typhimurium SE10/72, and *Campylobacter* jejuni RC039 with 0.125% As, 0.5% As and 50 or 200 μg/mL inulin. The controls included: (A) *F. prausnitzii* only; (B) *F. prausnitzii* + 50 μg/mL inulin; (C) *F. prausnitzii* + 200 μg/mL inulin; (D) *F. prausnitzii* + 0.125% AuraShield; (E) *F. prausnitzii* + 0.5% AuraShield; (F) *F. prausnitzii* + 200 μg/mL inulin + *C. jejuni*; (G) *F. prausnitzii* + 50 μg/mL inulin + *C. jejuni*; (H) *F. prausnitzii* + 0.125% AuraShield + *C. jejuni*; (I) *F. prausnitzii* + 0.5% AuraShield + *C. jejuni*; (J) *F. prausnitzii* + 50 μg/mL inulin + *S.* Typhimurium; (L) *F. prausnitzii* + 200 μg/mL inulin + *S.* Typhimurium; (M) *F. prausnitzii* + 0.125% AuraShield + *S.* Typhimurium; (N) *F. prausnitzii* + 0.5 % AuraShield + *S.* Typhimurium. The caeca were then closed with surgical string, suspended in a flask of DMEM, and incubated at 42 °C under microaerophilic conditions for 24 h. At the end of the 24 h incubation, the caecal contents were harvested for microbiological and butyrate levels measurements. The inulin concentrations were used as previously suggested (Babu et al., 2012).

### Butyrate measurements

Butyrate measurements were performed as previously described (Butucel et al., 2022), following the experimental design for the caecal *ex vivo* incubation model (Fig. 1.2). To quantify butyrate production, the contents were homogenised and stored at −20 °C until processing. Prior to measurement, they were diluted 1:4 in sterile water and homogenised in a stomacher (Seward, Premier, Scientific, UK). All experiments and measurements were performed in triplicate. Butyrate production was measured by GC-MS (SCION-456-GC) as previously described (Fagundes et al., 2021).

### Quantitative real-time PCR (qRT-PCR)

The mRNA expression levels of *CXCLi1, CXCLi2* and *ROMO*1 in the *ex vivo* caecal tissue were measured as previously described with small modifications after 24 h incubation. Each experiment was performed in triplicate and included for each investigated gene a standard Log10 dilution series of RNA form neutral tissue RNA (non-infected) for calibration (10^−1^-10^−5^) with a PCR efficiency of 1.89-1.92. Data were analysed as previously described and based on the 28S reference gene (Table 1) (Setta et al., 2012). Briefly, RNA from caeca tissue was extracted using the RNeasy Plus Mini Kit (Qiagen, United Kingdom). The RNA was reverse transcribed using Transcriptor First Strand cDNA Synthesis Kit (Roche) according to the manufacturer’s protocol. The mRNA levels were determined by quantitative RT-PCR using QuantiNova SYBR Green PCR Kit (Qiagen, United Kingdom) on a LightCycler 96 (Roche). The qPCR conditions included an initial heating step to 95 °C for 1 min, followed by 40 cycles of 95 °C for 20 s and 60 °C for 1 min. Relative gene expression levels were evaluated using the 2 -^ΔΔCT^ method with β-actin. The following cycle profiles were used: one cycle at 95 °C for 10 min; 45 cycles at 95 °C for 10 s, 60 °C for 30 s, and 72 °C for 1 s; and one cycle at 40 °C for 30 s. For quantification of *butCoA* expression, the bacterial cells followed the current protocol: to lyse the cells, 350 µL of Buffer RLT Plus (RNeasyPlus Mini Kit; Qiagen, Manchester, UK) was added to each pellet, vortexed for 30 s, and sonicated using an ultrasonic processor for 2–3 s at 70% amplitude. The sonicator metal rod was washed with RNaseZAP™ (Thermo Fisher, Horsham, UK) to prevent contamination of the samples. Total RNA was isolated following the manufacturer’s protocol using an RNeasyPlus Mini Kit (Qiagen, Manchester, UK). The purity of the RNA isolates was measured using a NanoDrop 1000 UV/VIS Spectrophotometer (Thermo Fisher, Horsham, UK). The following thermal profile was used: 2 min at 50 °C, 10 min at 95 °C, and 40 cycles of 15 s at 95 °C and 60 s at 60 °C. The *efp* gene, encoding the translation elongation factor P, was used for normalisation. Primers used in this study are shown in Table 1.

**Table 1:** Primers used in this study.

Target gene	P/P	Sequence	Accession number
*28S*	P	5′-(FAM)-aggaccgctacggacctccacca-(TAMRA)-3′	X59733
	F	5′-ggcgaagccagaggaaact-3′	
	R	5′-gacgaccgatttgcacgtc-3′	
*CXCLi1*	P	5′-(FAM)-ccacattcttgcagtgaggtccgct-(TAMRA)-3′	AF277660
	F	5′-ccagtgcatagagactcattccaaa-3′	
	R	5′-tgccatctttcagagtagctatgact-3′	
*CXCLi2*	P	5′-(FAM)-tctttaccagcgtcctaccttgcgaca-(TAMRA)-3′	AJ009800
	F	5′- gccctcctcctggtttcag-3′	
	R	5′-tggcaccgcagctcatt-3′	
*ROMO1*	F	5′-agcccagctgcttcgacagagt-3′	NM_001396978.1
	R	5′-cgtcctctcatgccgatcctga-3′	
*butCoA*	F	5′-actttgttctgggcgcatac-3′	CG447_01820
	R	5′-ggtcagtcccttcaggttca-3′	
*efp*	F	5′-gttgagttccagcacgtgaa-3′	CG447_05125
	R	5′-aaagcctgagggaactttgc-3′	
*β-actin*	F	5′-ctggcacctagcacaatgaa-3′	NM_205518.2
	R	5′-acatctgctggaaggtggaccat-3′	

P/P – Primer/Probe.

### Statistical methods

Statistical analyses were performed using GraphPad software, version 11. In some cases, data were represented as mean ± SD. *P*-values < 0.05 were considered statistically significant. One-way ANOVA, Two-way ANOVA and Dunnett’s multiple comparisons tests were used depending on the power needed. Data distribution was assessed using the Shapiro–Wilk test to verify normality. Homogeneity of variances was evaluated using Levene’s test. Biological replicates are defined as independent bacterial cultures or independent caecal culture experiments (n = 3) performed on different days. Technical replicates are defined as multiple measurements (e.g., replicate wells or readings) from the same biological sample.

## Results

### Subinhibitory dose of AuraShield enhances butyrate release by F. prausnitzii-mediated inhibition of Salmonella and Campylobacter

**Fig. 2:**
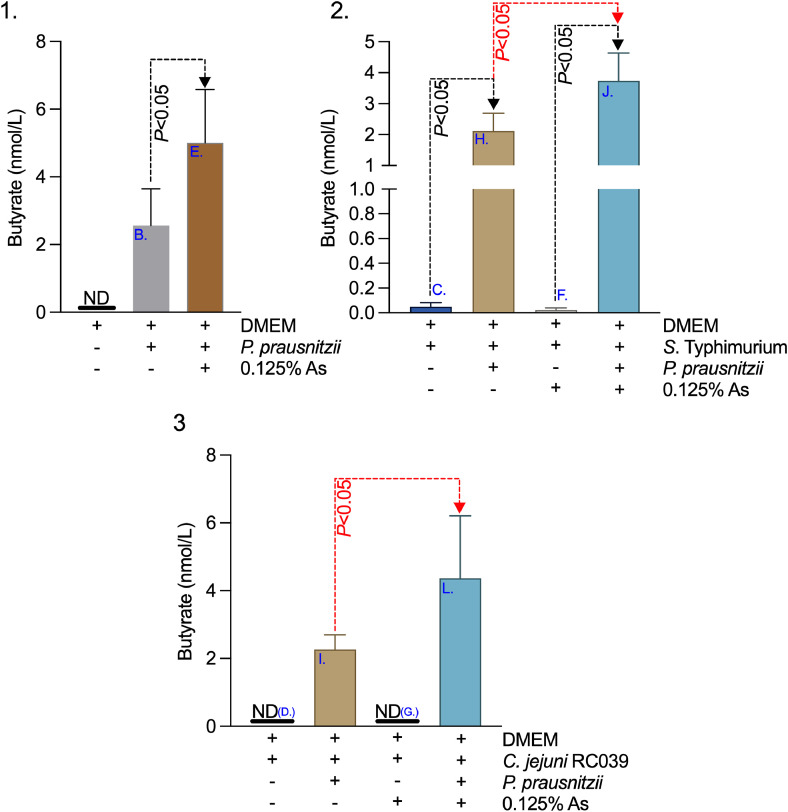
Subinhibitory 0.125% As enhances *F. prausnitzii*‑mediated inhibition of *S.* Typhimurium and *C. jejuni* RC039 and promotes butyrate production in the *ex vivo* chicken caecal model. Butyrate concentrations in caecal contents were measured by gas chromatography. (Figure 2.1) Butyrate levels during *F. prausnitzii* growth in the presence or absence of 0.125% As; (Figure 2.2) butyrate levels and during co‑culture of *F. prausnitzii* with *S*. Typhimurium SE 10/72 in the presence of 0.125% As; (Figure 2.3) butyrate levels and during co‑culture of *F. prausnitzii* with *C. jejuni* RC039 in the presence of 0.125% As. Data are presented as mean ± SD from three independent experiments. Statistical significance was assessed using one‑way ANOVA with Bonferroni’s post‑hoc test; *P* < 0.05 was considered statistically significant.

Firstly, we have determined the MIC and MBC concentrations at which AuraShield affects the growth of *C. jejuni* RC039 and *S*. Typhimurium SE10/72. For *C. jejuni* RC039, the MIC was 0.25% As and the MBC was 0.5% As. For *S*. Typhimurium SE10/72, the MIC was 0.5%, whereas the MBC was 1%. Based on these results, the subinhibitory concentration of 0.125% As was chosen for further experiments. Secondly, we have designed an *in vitro* organ culture model to test whether As can act as a prebiotic to stimulate the growth of *F. prausnitzii* and to impede the growth of *S*. Typhimurium SE 10/72 and *C. jejuni* RC039 (Fig. 1.1 and 1.2). In this caecal model, incubation of whole caeca for 24 h at 42 °C under microaerophilic conditions allowed simultaneous assessment of the effects of 0.125% As on *F. prausnitzii* and its ability to limit colonisation by *S*. Typhimurium SE10/72 and *C. jejuni* RC039 (Fig. 1.3). In caeca inoculated with *F. prausnitzii* alone, bacterial levels increased after 24 h, as shown in Fig. 1.4 (from a mean of 6 log₁₀ CFU/mL to 11 log₁₀ CFU/mL; *P* < 0.05). In the absence of *F. prausnitzii, S*. Typhimurium (5 log₁₀ CFU/mL) and *C. jejuni* (3.5 log₁₀ CFU/mL) established robust colonisation in the caecal model. Co‑incubation with *F. prausnitzii* significantly reduced pathogen loads compared with pathogen‑only controls. Separate exposure to 0.125% As and *F. prausnitzii* significantly reduced the levels of *S*. Typhimurium (Fig. 1.5) and *C. jejuni* (Fig. 1.6), and this reduction was further enhanced when As and *F. prausnitzii* were used simultaneously (*P* < 0.05). Collectively, these data indicate that 0.125% As stimulated *F. prausnitzii* growth and exerts a stronger inhibitory effect on *S*. Typhimurium and *C. jejuni*. The combination of As and *F. prausnitzii* produces additive or synergistic reductions in pathogen counts. Butyrate concentrations in the *ex vivo* caecal model are shown in Fig. 2. In caeca inoculated with *F. prausnitzii* alone (Fig. 2.1), butyrate levels increased over 24 h, and this increase was further enhanced in the presence of 0.125% As, indicating that subinhibitory As can stimulate butyrate production by this commensal. When *F. prausnitzii* was co‑cultured with *S*. Typhimurium SE10/72 (Fig. 2.2) or *C. jejuni* RC039 (Fig. 2.3), butyrate levels remained elevated compared with pathogen‑only conditions, and the addition of 0.125% As led to a further rise in butyrate concentrations.

### Upregulation of butCoA and increased butyrate production by F. prausnitzii in response to AuraShield

To further confirm our hypothesis that the organic acid blend stimulates and increases the production and release of butyrate by *F. prausnitzii*, we have designed an experimental protocol (Fig. 3.1) to measure the bacterial *butCoA* expression and the released butyrate levels. The conventional culture experimental design is presented in Fig. 3.1. The results clearly show a significant increase in the *butCoA* gene expression (*P* < 0.05) at both 0.125% and 0.5% As during 48h of growth under microaerophilic conditions (Fig. 3.2). The observed increase in *butCoA* gene expression was also accompanied by a significant increase (*P* < 0.05) in butyrate release by *F. prausnitzii* in the culture supernatant in the presence of 0.125% or 0.5% As (Fig. 3.3). These findings provide further support for the proposed stimulatory effect of As on the butyrogenic activity of *F. prausnitzii*. Notably, increased *butCoA* expression was associated with higher extracellular butyrate concentrations.

**Fig. 3:**
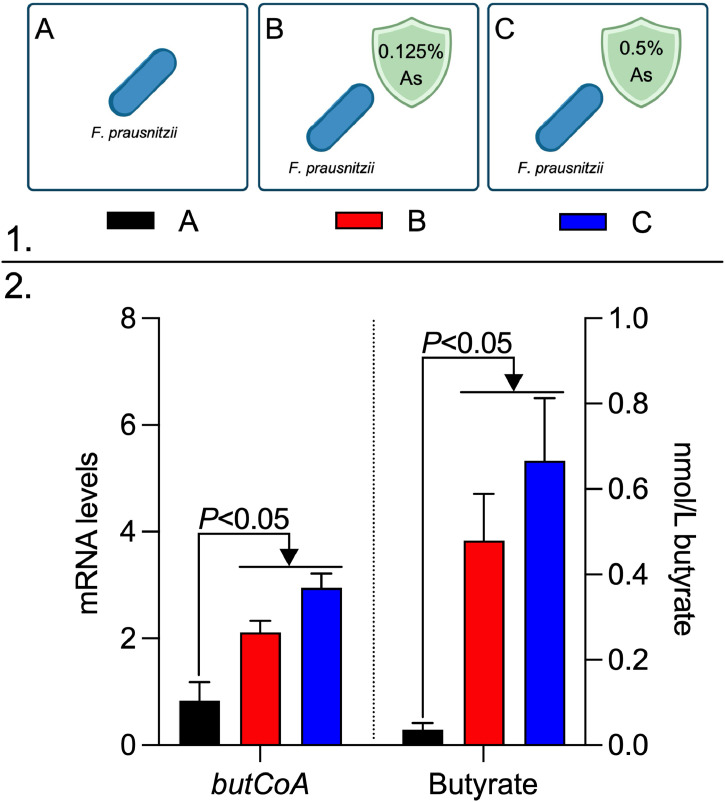
AuraShield upregulates *butCoA* expression and enhances butyrate production by *F. prausnitzii* under conventional culture conditions. *F. prausnitzii* was grown under microaerophilic conditions in the presence or absence of AuraShield (As) at 0.125% or 0.5% for 48 h. (1) Schematic representation of the conventional culture experimental design. (2) Relative expression of the *butCoA* gene and butyrate concentrations in culture supernatants measured by gas chromatography. Data are presented as mean ± SD from three independent experiments. Statistical significance was assessed using one-way ANOVA with Bonferroni’s post hoc test; *P* < 0.05 was considered statistically significant.

### The comparative effects of AuraShield and inulin control on F. prausnitzii butyrate production during conventional multi-bacterial co-culture

We have next investigated the effect of the subinhibitory As concentration of 0.125% on *F. prausnitzii* butyrate production during co-culture with conventional bacteria (*C. jejuni* RC039 and *S*. Typhimurium SE10/72). However, in this instance, the effect of the well-known butyrate inducer, inulin (50 and 200 µg/mL), was compared with the sub-inhibitory concentration of 0.125% As and with the MIC of 0.5% As (Fig. 4.1). Both inulin and As significantly increased butyrate production in *F. prausnitzii* only culture, with concentrations of 0.125% and 0.5% As producing effects comparable to those observed at 50 or 200 µg/mL inulin (Fig. 4.2A–4.2E). During co‑culture of *F. prausnitzii* with *C. jejuni* (Fig. 4.2F–4.2I) or *S*. Typhimurium (Panels J–N), the As or inulin effect on butyrate formation was not affected by the pathogen presence. Butyrate formation was clearly As- and inulin concentration-dependent, with 0.5% As and 200 µg/mL inulin significantly (*P* < 0.05) more efficient at inducing butyrate formation by *F. prausnitzii* than 0.125% As or 50 µg/mL inulin (Fig. 4.2).

**Fig. 4:**
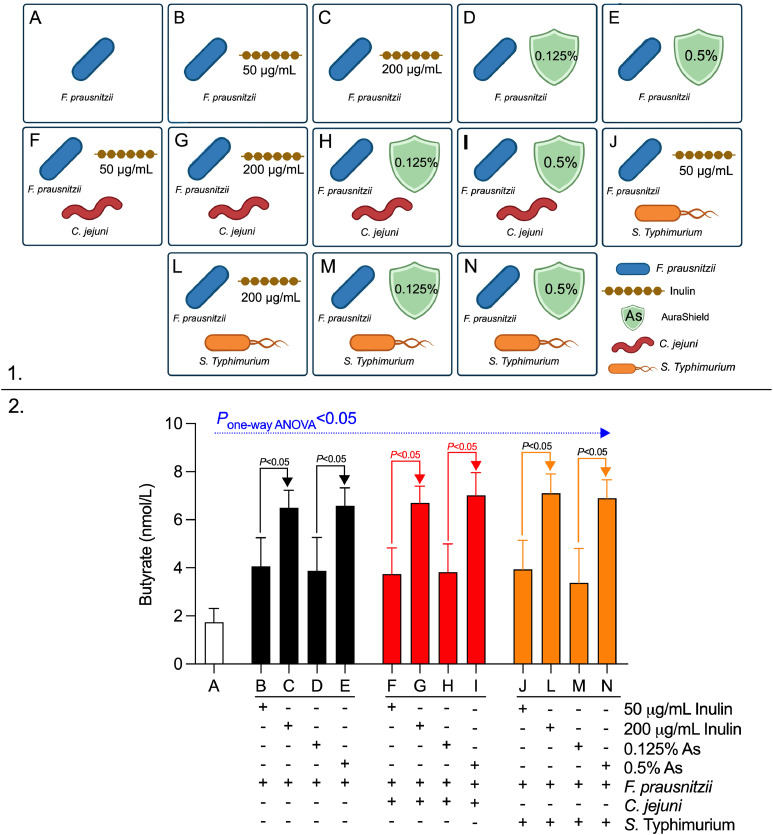
Comparative effects of As (0.125% and 0.5%) and the inulin control (50 and 200 µg/mL) on butyrate production during co-culture of *F. prausnitzii* with *C. jejuni* RC039 and *S*. Typhimurium SE10/72. Figure 4.1 illustrates the co‑culture combinations (1A–1N). Figure 4.2 presents butyrate levels as the mean ± SD from three independent experiments. Statistical significance was assessed using one‑way ANOVA with Bonferroni’s post‑hoc test; *P* < 0.05 was considered statistically significant.

### Subinhibitory AuraShield modulates the inflammatory chemokine expression of CXCLi1 and CXCLi2 in the ex vivo chicken caecal co-incubation model

**Fig. 5:**
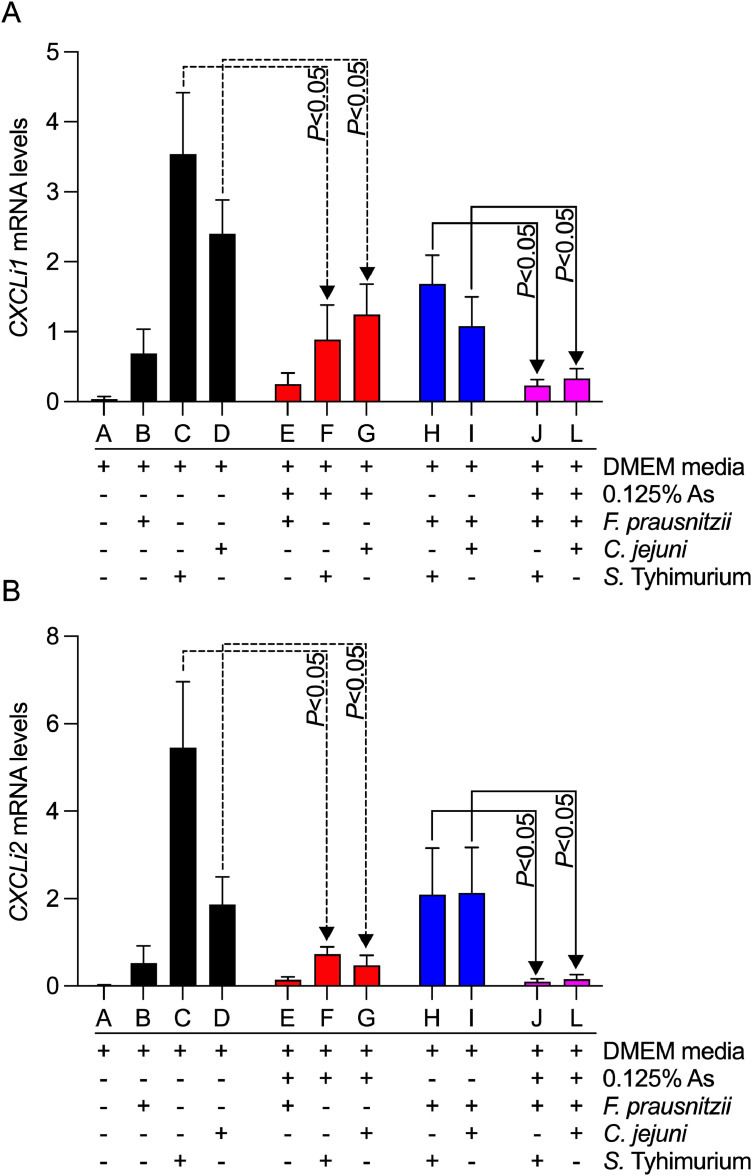
*CXCLi1* and *CXCLi2* chemokine gene expression levels in the *ex vivo* chicken caeca co-incubation model. (A) *CXCLi1* mRNA gene expression levels; (B) *CXCLi2* mRNA gene expression levels. Data from three independent experiments are presented. Statistical significance was assessed using one‑way ANOVA with Bonferroni’s post‑hoc test; *P* < 0.05 was considered statistically significant.

The relative mRNA expression levels of *CXCLi1* (Fig. 5A) and *CXCLi2* (Fig. 5B) were measured in chicken caecal tissue following 24 h incubation at 42 °C under microaerophilic conditions in the *ex vivo* model containing *F. prausnitzii*, S. Typhimurium SE10/72, and/or *C. jejuni* RC039 in the presence or absence of 0.125% As), as indicated in the experimental design (Fig. 1.2). The relative mRNA expression levels of *CXCLi1* and *CXCLi2* in *ex vivo* chicken caecal tissue were significantly reduced (*P* < 0.05) after 24 h of co-incubation with *F. prausnitzii, S*. Typhimurium SE10/72, and/or *C. jejuni* RC039, with or without 0.125% AuraShield.

### AuraShield, in combination with F. prausnitzii, further enhances the antioxidant effect ex vivo

**Fig. 6:**
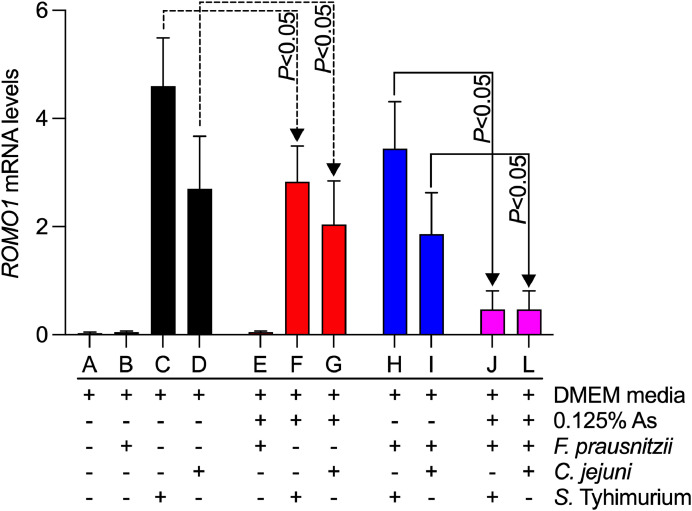
ROMO1 mRNA expression levels in the *ex vivo* chicken caeca co-incubation model. Data from three independent experiments are presented. Statistical significance was assessed using one‑way ANOVA with Bonferroni’s post‑hoc test; *P* < 0.05 was considered statistically significant.

To investigate whether the observed effects of *F. prausnitzii* and AuraShield extended to oxidative stress-related responses in the chicken caecal model, *ROMO1* mRNA expression was measured in *ex vivo* caecal tissue after 24 h incubation under microaerophilic conditions. As shown in Fig. 6, co-incubation with *F. prausnitzii* in the presence of 0.125% AuraShield reduced ROMO1 expression (*P* < 0.05) compared with the relevant pathogen-only and untreated control groups. This effect was observed with *S*. Typhimurium SE10/72 and *C. jejuni* RC039, indicating that the combination of AuraShield and *F. prausnitzii* could be associated with a lower oxidative stress response in infected caecal tissue.

### Pathogen growth inhibition under conventional co-culture is comparable between AuraShield and inulin

To verify if increased butyrate production also translates into pathogen growth inhibition, we designed an experiment to investigate whether the observed increase in butyrate production during conventional growth of *F. prausnitzii,* or in co-culture with *C. jejuni* RC039 and *S*. Typhimurium SE10/72. To demonstrate this effect, we chose the subinhibitory concentration of 0.125% As and the lowest inulin concentration of 50 µg/mL to minimise interference with pathogen survival (Fig. 7.1). Under control conditions, *S*. Typhimurium SE10/72 and *C. jejuni* RC039 achieved high growth levels after 24 h incubation (Fig. 7.2). The addition of 0.125% As significantly reduced the counts of both *S*. Typhimurium and *C. jejuni* compared with untreated controls (*P* < 0.05). Inulin at 50 µg/mL also reduced pathogen loads, confirming its established prebiotic function (*P* < 0.05). The magnitude of reduction observed with 0.125% As was comparable to that observed with inulin.

**Fig. 7:**
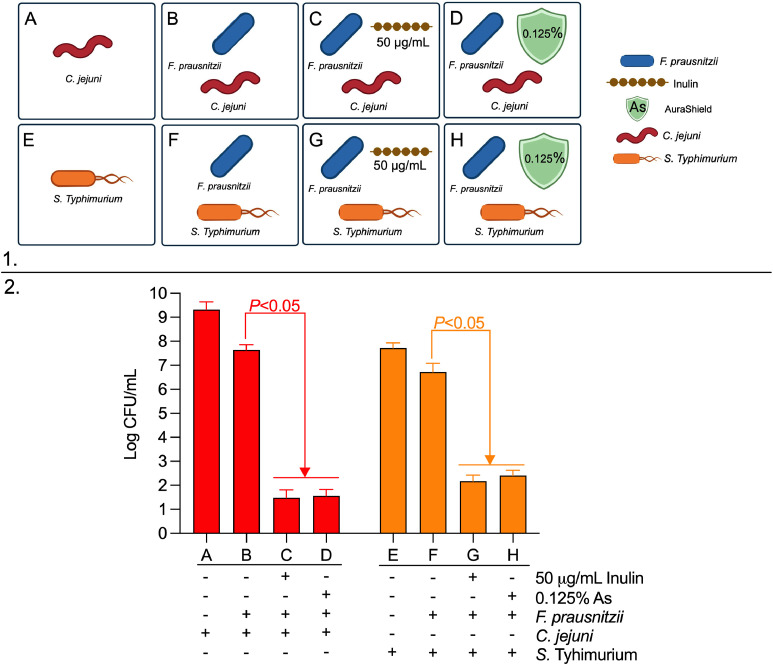
Comparative prebiotic effects of subinhibitory AuraShield (As) and inulin on *F. prausnitzii*, pathogen exclusion during conventional growth. *S*. Typhimurium SE10/72, and *C. jejuni* RC039, were incubated for 24 h at 42 °C under microaerophilic conditions in the presence or absence of 0.125% As and inulin (50 μg/mL), as indicated. Figure 7.1 shows the experimental design. Figure 7.2 shows the *S*. Typhimurium SE10/72, and *C. jejuni* RC039 counts as impacted by 0.125% As and 50 μg/mL inulin. Data are presented as mean ± SD from three independent experiments. Statistical significance was assessed using one‑way ANOVA with Bonferroni’s post‑hoc test; P < 0.05 was considered statistically significant.

## Discussion

The strategy of utilising natural antimicrobial mixtures fortified with organic acids and botanical extracts offers significant promise for effective pathogen management (Vaou et al., 2021). In an earlier investigation, a natural antimicrobial mixture comprising organic acids and citrus and olive extracts was assessed for its anti-staphylococcal activity (Balta et al., 2024) in a mastitis model. In this study, blends of organic acids exerted a multifaceted effect during the infection process. Initially, it mitigated bacterial virulence attributes, such as exopolysaccharides (EPS) and biofilm, while concurrently compromising bacterial membrane integrity. Further, this mixture hindered the adhesion of *S. aureus* DSM1104 to epithelial cells, bolstered epithelial tight junctions, and did so without compromising cellular viability. Moreover, it notably diminished inflammation and curbed oxidative stress in the infected cells. This antioxidative property can be attributed to its inhibition of extracellular signal-regulated kinases (ERKs), key pathways that govern cellular resilience during oxidative challenges and orchestrate the immune response via oxidative dephosphorylation.

Establishing and using subinhibitory concentrations of antimicrobials is essential as it allows investigations on bacterial pathogens’ morphology and metabolism (Derakhshan et al., 2008). The present preliminary results demonstrate that a subinhibitory concentration of AuraShield (As) could favour the growth and metabolic activity of *F. prausnitzii,* thereby potentially competitively excluding certain bacterial pathogens, including *S*. Typhimurium and *C. jejuni*. MIC and MBC data confirmed that the selected concentration of 0.125% As was below the inhibitory threshold for both pathogens, indicating that the observed effects in the *ex vivo* caecal model were not solely due to direct bactericidal activity. Instead, these results suggest that low-dose As may exert a selective ecological effect, promote beneficial commensal activity and indirectly enhance resistance to pathogen establishment (Yoon et al., 2024). A key finding of this study was also that *F. prausnitzii* proliferated in the caecal model over the 24 h incubation period, and that this response was enhanced in the presence of 0.125% As. This finding supports the hypothesis that As may possess prebiotic-like properties under these experimental conditions (Waghmare et al., 2025). Given the well-established role of *F. prausnitzii* as a major butyrate-producing commensal with anti-inflammatory and barrier-supporting functions, stimulating its growth may have important implications for gut health in both monogastric (piglets) and ruminants (calves) (Foditsch et al., 2014). The concurrent increase in butyrate concentrations further strengthens this interpretation and indicates that As not only supports the growth of probiotic-like bacteria but can also potentially play a role in the treatment and prevention of various diseases and in health promotion (Yadav et al., 2022). Additionally, we showed that the role of the organic mixture (As) in increasing butyrate formation *ex vivo* is regulated by the expression of the butCoA gene involved in butyrate formation in *F. prausnitzii* via the utyryl-CoA:acetate CoA-transferase pathway (Verstraeten et al., 2024).

In chickens, *Salmonella* infection and colonisation of the caecum start with an inflammatory response that includes overexpression of the *CXCLi1* and *CXCLi2* chemokines (Wigley, 2014). The expression of these chemokines is enhanced during *Salmonella* infection (Cheeseman et al., 2008). In our caecal chicken infection model, the inclusion of the subinhibitory concentration of As (0.125%) during co-infection with *F. prausnitzii, C. jejuni* RC039 and *S*. Typhimurium SE10/72 led to a significant downregulation of both chemokines. This effect was also mirrored in the conventional co-culture model, alongside increased butyrate production and lowered pathogen presence observed in both models.

Oxidative stress, arising from an imbalance between reactive oxygen species (ROS) and weak antioxidant defences, leads to impaired animal health, compromised immunity, and reduced productivity (Khan et al., 2025). *ROMO1* is a vital enzyme in the chicken caecum that regulates reactive oxygen species production and controls the redox signalling during infection (Lee et al., 2018). Interestingly, our preliminary evidence suggests that *ROMO1* was downregulated in caecal tissue during infection with *C. jejuni* RC039 and *S*. Typhimurium SE10/72 in the presence of 0.125% As and *F. prausnitzii*. A similar mechanism, involving the downregulation of *ROMO1* expression, was described in the caeca of chickens fed with organic acids (oleanolic acid), alongside a positive effect on the abundance of *F. prausnitzii* in the caecum (Tu et al., 2024). The chemokine expression data also suggest that AuraShield may influence host-pathogen-microbiota interactions beyond bacterial enumeration. *CXCLi1* and *CXCLi2* are key chemokines that mediate heterophil recruitment and early inflammatory responses to enteric infection (De Filippo et al., 2013). Although the present study was not designed to fully resolve the immunomodulatory pathways involved, modulation of these markers in *ex vivo* caecal tissue indicates that AuraShield can influence the mucosal inflammatory environment. This is important because excessive inflammation may favour pathogen expansion and tissue damage, whereas a more controlled response may support colonisation resistance and epithelial homeostasis (Ducatelle et al., 2023). Further work will be required to determine whether the observed chemokine modulation is due to reduced pathogen burden, increased butyrate production, direct host signalling by AuraShield components, or a combination of these factors.

Inulin is primarily recognized for its prebiotic and dietary fibre properties, supporting the growth of beneficial gut microbiota and improving digestive health (Qin et al., 2023). While inulin mainly contributes to gut health, AuraShield may exert a stronger effect on protection or performance (Balta et al., 2021). The comparison suggests that inulin could offer physiological benefits, whereas AuraShield may act more directly on controlling the persistence of certain bacterial pathogens. Therefore, the relative effect of each ingredient depends on the desired outcome: inulin may be more effective where digestive or prebiotic benefits are the priority (Bucław, 2016), while AuraShield may be more advantageous where enhanced protection.

**Fig. 8:**
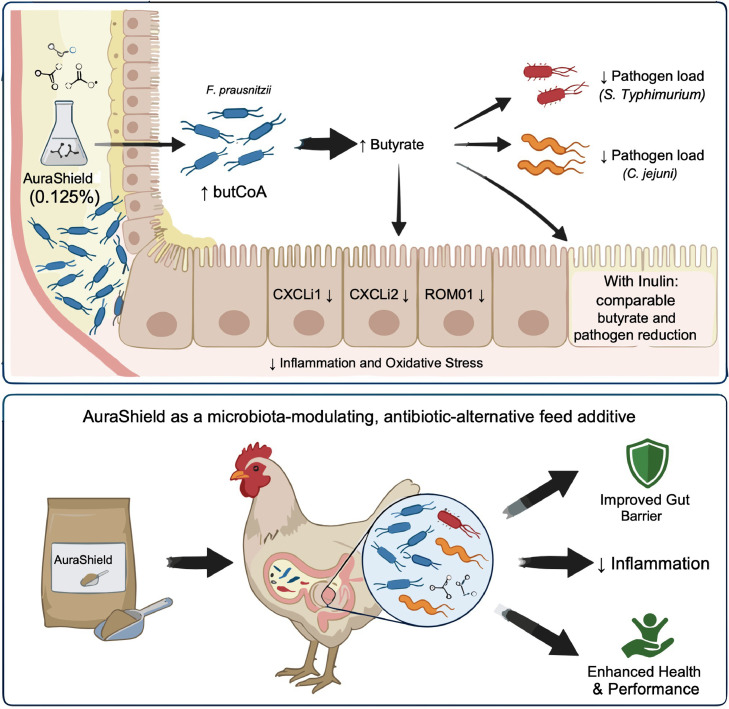
summarises the study’s overall conclusion. In the ex vivo chicken caecal model, a subinhibitory concentration of AuraShield (0.125%) created a more favourable intestinal environment by promoting the growth and metabolic activity of *Faecalibacterium prausnitzii*. This was accompanied by increased butyrate production, indicating enhanced beneficial microbial function. At the same time, AuraShield reduced colonisation by *Salmonella Typhimurium* and *Campylobacter jejuni*, particularly when combined with *F. prausnitzii*, suggesting an additive or synergistic protective effect. Beyond its antimicrobial and microbiota-modulating actions, AuraShield was also associated with reduced expression of the inflammatory chemokines *CXCLi1* and *CXCLi2* and the oxidative stress marker *ROMO1*. Overall, Fig. 8 illustrates that AuraShield acts not only as an antimicrobial additive but also as a functional microbiota-modulating agent with prebiotic-like effects that may help improve poultry gut health and colonisation resistance.

This study demonstrates that a subinhibitory concentration of AuraShield (0.125%) can beneficially modulate the chicken caecal environment in an *ex vivo* model. At this dose, AuraShield enhanced the growth of *Faecalibacterium prausnitzii* and increased butyrate production, while simultaneously reducing colonisation by *S.* Typhimurium and *Campylobacter jejuni*. These effects were further strengthened when AuraShield was combined with *F. prausnitzii*, suggesting an additive or synergistic effect between the organic acid blend and this beneficial commensal bacterium. In addition to its effects on bacterial dynamics, AuraShield was associated with reduced expression of the inflammatory chemokines *CXCLi1* and *CXCLi2*, as well as the oxidative stress marker *ROMO1*, indicating a broader protective effect on the host mucosal environment (Fig. 8). Overall, these findings support the view that subinhibitory AuraShield could potentially act as a prebiotic-like supplement, however, more in vivo testing is necessary. We would like to emphasise that limitations are associated with any *ex vivo* models, and our design is also subject to such assumptions. That is why further *in vivo* studies are warranted to confirm these effects under commercial production conditions and to determine the mechanisms underlying its interaction with host tissues and the gut microbiota.

## CRediT authorship contribution statement

**Ana-Maria Imbrea:** Investigation, Formal analysis, Conceptualization. **Igori Balta:** Investigation, Formal analysis, Data curation. **Lavinia Stef:** Resources, Project administration, Formal analysis. **Todd Callaway:** Writing – original draft, Methodology, Conceptualization. **Ioan Pet:** Writing – review & editing, Writing – original draft, Supervision, Conceptualization. **Nicolae Corcionivoschi:** Writing – review & editing, Writing – original draft, Project administration, Funding acquisition, Formal analysis, Conceptualization.

## Disclosures

The author(s) declare(s) no conflicts of interest.

## Data Availability

Data can be made available on request.
